# Remission of Symptoms of Functional Neurological Disorder (FND) Utilising Novel Interventions: A Case Report

**DOI:** 10.1192/bjo.2024.686

**Published:** 2024-08-01

**Authors:** Roopa Rudrappa, Roni Altman, Mohanbabu Rathnaiah

**Affiliations:** 1 Derbyshire Health Care NHS Foundation Trust, Nottingham, United Kingdom; 2 University of Nottingham, Nottingham, United Kingdom; 3 Derbyshire Health Care NHS Foundation Trust, Derby, United Kingdom; 4 Institute of Mental Health, University of Nottingham, Nottingham, United Kingdom

## Abstract

**Aims:**

FND can be considered as an umbrella term that includes range of motor and sensory system dysfunctions with genuine symptoms including paralysis, tremors, sensory disturbance, speech disturbance and seizure. Functional seizures usually termed as Non-Epileptic attack disorder (NEAD) can result in profound persisting disability. Brief bouts of unprovoked and uncontrollable laughter, spontaneous in origin, combined with facial contraction in the form of smile, is termed as ‘gelastic seizures’. Modafinil is a dopamine modulating molecule for which evidence is accumulating towards its cognitive enhancement role in multiple domains. Furthermore, it has been shown to promote hippocampal neurogenesis and synaptic plasticity in pre-clinical studies. We report a case of FND in which pharmacological (Modafinil) and non-pharmacological interventions (Brain retraining) resulted in resolution of symptoms of probable gelastic episodes.

**Methods:**

A 50-year-old lady who was referred by consultant neurologist to our Neuropsychiatry pilot service with episodes of uncontrollable laughing, singing, screaming and suffering from staggering and imbalance. Following these episodes, patient described sleeping for hours to days with fatigue. Her husband first noticed low mood 12 years ago during post-natal period. Treatment with fluoxetine reportedly contributed to ‘cyclical highs and mood variations’. One year later, her ‘gelastic episodes’ started and continued to occur every 2 or 3 months and they were brought on by a range of factors including tiredness, menstrual periods and stress. Patient also reported atypical cognitive deficits such as ‘losing vocabulary’ and ‘stuck every couple of seconds’. Furthermore, detailed history confirmed possible traits of attention deficit and hyperactive thinking since her childhood.

**Results:**

Following a comprehensive assessment, the role of the brain in the manifestation of her symptoms was discussed and agreed upon. Strategies based on Cognitive Behaviour Therapy principles such as active distraction, brain retraining, engaging in therapeutic activities and expressive writing were discussed and agreed upon. Following detailed risk-benefit analysis, modafinil was initiated at 200 mg dose in the morning. Patient made a remarkable recovery nearly back to her baseline with resolution of her gelastic episodes and thus improvement in her mental state. She continues to be stable in the community.

**Conclusion:**

This case highlights the importance of recognising and treating cluster of symptoms which might belong to the impulsive-compulsive spectrum. This further emphasises the role of dopamine-modulating agents such as modafinil along with brain-retraining strategies.

